# Progress of Medical Science in Germany

**Published:** 1875-01-15

**Authors:** Edmund J. Doering


### PROGRESS OF MEDICAL SCIENCE IN GERMANY.

By Edmund J. Doering, M.D.

I. Treatment of Scabies, by Dr. Clemens (*Allg. Med. Central Zeitung*, No. 98). II. Treatment of Epididymitis, by Dr. Calvo (*Allg. Wiener Med. Z.* No. 49). III. Treatment of Whooping-Cough, by Dr. Mascarel (*Med. Chir. Rundschau*, IV B., I Heft).

I.

DR. CLEMENS, on the basis of repeated and careful experiments, considers the following prescription for Scabies as not only more convenient for general use than the numerous remedies now employed, but also as more efficacious:

| | | |
|---|---|---|
| ℞ | Acid Arseniosi. | gr. j. |
| | Potass. Carbonate, | gr. xv. |
| | Spirit Sapon. | f ℥ ss. |
| | Aqu. Font. | f ℥ iij.—M. |

The carbonate of potassa is added to increase the solubility of the arsenious acid, and also to prevent the too rapid drying of the lotion when applied to the skin. One-tenth part of this mixture is a sufficient quantity for each application, and the patient is directed to rub it well into the skin, at least twice daily. This lotion is quite harmless, for the quantity of arsenic absorbed by the skin is too minute to justify any fears of poisoning, the author having used it even for young children without producing any ill effects, not even derangement of the digestive organs.

II.

This frequent complication of Gonorrhœa is best treated on the following plan: First, apply from ten to twenty leeches to the perineum along the affected side, covering the testicles constantly with warm poultices, to which twenty drops of laudanum may be added. Absolute rest in bed, and abstinence from all stimulating food must be strictly enjoined. Oatmeal gruel forms the best drink for this class of cases. Every other morning the patient is directed to drink two glasses of seidlitz-water. At a later stage of this affection, frictions of the testicles are to be made thrice daily with the following salve:

| | | |
|---|---|---|
| ℞ | Ext. Bellad. | ʒ iss. |
| | Ung. Hydr. | ℥ j.—M. |

If hydrocele has developed, causing severe pain, small punctures into the tunica vaginalis with a sharp lancet will be of use. After the inflammation has subsided, and only a swelling of the testicles remains, frictions with the following ointment will be found to be very useful:

| | | |
|---|---|---|
| ℞ | Ext. Bellad. | ʒ iss. |
| | Potass.-iodidi, | ʒ j. |
| | Adipis. | ℥ j.—M. |

The scrotum must be wrapped in cotton and be constantly supported by a suspensory bag. If the swelling persists and seems to depend on the condition of the general constitution, then gentian with iodine, cod-liver oil, quinine, salt-baths, etc., will be indicated. Emissions of bloody semen becoming permanent, point to an inflammation of the vesiculæ seminales, and therefore demand a separate treatment of these organs.

III.

Dr. M. recommends the following treatment for Whooping-cough:

1. Every morning between five and eight o'clock, the patient receives from a teaspoonful to a tablespoonful, according to age, of a solution of tartar emetic (1 gr. to 4 oz. water). Sensitive children receive ipecac instead.

2. After supper, one-sixth of grain of extract of belladonna is given, increasing the dose gradually to one grain. After the paroxysms diminish in frequency, the dose of the belladonna may be diminished correspondingly. The extract must be pure and produce the characteristic erythema and dryness of the throat. If the general nutrition of the little patient suffers from too frequent vomiting, small doses of morphine may be given every three or four hours, combined with the administration of five or six teaspoonfuls of black coffee after breakfast.

The author has followed this plan of treatment for 18 years, and has invariably diminished the duration o the disease to four and three weeks and even less.

